# Respiratory processes in non-photosynthetic plastids

**DOI:** 10.3389/fpls.2015.00496

**Published:** 2015-07-17

**Authors:** Marta Renato, Albert Boronat, Joaquín Azcón-Bieto

**Affiliations:** ^1^Departament de Biologia Vegetal, Facultat de Biologia, Universitat de Barcelona, Barcelona, Spain; ^2^Centre de Recerca en Agrigenòmica, Consorci CSIC-IRTA-UAB-UB, Campus Universitat Autònoma de Barcelona, Bellaterra, Spain; ^3^Departament de Bioquímica i Biologia Molecular, Facultat de Biologia, Universitat de Barcelona, Barcelona, Spain

**Keywords:** plastid, chromoplast, etioplast, amyloplast, chlororespiration, chromorespiration, PTOX, plastid respiration

## Abstract

Chlororespiration is a respiratory process located in chloroplast thylakoids which consists in an electron transport chain from NAD(P)H to oxygen. This respiratory chain involves the NAD(P)H dehydrogenase complex, the plastoquinone pool and the plastid terminal oxidase (PTOX), and it probably acts as a safety valve to prevent the over-reduction of the photosynthetic machinery in stress conditions. The existence of a similar respiratory activity in non-photosynthetic plastids has been less studied. Recently, it has been reported that tomato fruit chromoplasts present an oxygen consumption activity linked to ATP synthesis. Etioplasts and amyloplasts contain several electron carriers and some subunits of the ATP synthase, so they could harbor a similar respiratory process. This review provides an update on the study about respiratory processes in chromoplasts, identifying the major gaps that need to be addressed in future research. It also reviews the proteomic data of etioplasts and amyloplasts, which suggest the presence of a respiratory electron transport chain in these plastids.

## Introduction

Plastids likely originated through the process of endosymbiosis, which consisted in the integration of a free-living photosynthetic prokaryote into a eukaryotic cell. This prokaryote was probably an ancestor of current cyanobacteria and provided its host the capacity to obtain energy through oxygenic photosynthesis (Bédard and Jarvis, 2005; Gould et al., 2008). Beside chloroplasts, different types of non-photosynthetic plastids have evolved in plants, for instance chromoplasts, amyloplasts, and elaioplasts. These plastids carry out specialized functions in non-green tissues, mainly the biosynthesis and storage of carotenoids, starch and lipids, respectively (Whatley, 1978; Bédard and Jarvis, 2005; Li and Yuan, 2013).

In cyanobacteria, photosynthetic and respiratory chains are interconnected in the same membrane and share some electron carriers, like plastoquinone (PQ; Bennoun, 1982). Although the endosymbiosis event resulted in a reduction of the metabolic complexity of the free-living prokaryote (Gould et al., 2008), plastids could have retained some relics of its ancestral respiratory pathway (Cournac et al., 2000). The first experimental pieces of evidences confirming the existence of a respiratory chain in chloroplasts were provided by Bennoun (1982), who defined chlororespiration as a light-independent electron transport pathway from NAD(P)H to oxygen in thylakoid membranes. Afterward, the characterization of the *Arabidopsis thaliana* mutant *immutans* demonstrated that the oxidase responsible of the oxygen consumption in chlororespiration is the plastid terminal oxidase (PTOX), a monomeric oxidase similar to the mitochondrial alternative oxidase (Carol et al., 1999; Wu et al., 1999). Later studies indicated that the NAD(P)H-PQ oxidoreductase activity might be performed by the thylakoidal NAD(P)H dehydrogenase complex (Ndh; Burrows et al., 1998; Endo et al., 1998; Sazanov et al., 1998) or a type II NAD(P)H dehydrogenase (Desplats et al., 2009).

Chloroplast respiration has been extensively studied and reviewed (Peltier and Cournac, 2002; Rumeau et al., 2007; McDonald et al., 2011; Foudree et al., 2012; Nawrocki et al., 2015), but there is no consensus about its biological role. The most accepted hypothesis is that chlororespiration acts as a safety valve to prevent the over-reduction of the photosynthetic machinery in stress conditions (Laureau et al., 2013; Paredes and Quiles, 2013; Zivcak et al., 2013; Yu et al., 2014). Other proposed roles are photoprotection during dark to light transition (Joët et al., 2002) and balance the ATP/NADPH requirements in chloroplasts (Rumeau et al., 2005). However, the overexpression of PTOX does not result in higher photoprotection on photosystems in stress conditions or during acclimation (Rosso et al., 2006; Heyno et al., 2009). Moreover, chlororespiratory activity is very minor given that the electron flux through PTOX is always two orders of magnitude lower than through cytochrome b_6_f complex (Trouillard et al., 2012). On the other hand, it has been shown that PTOX has a dual role and also participates in carotenoid biosynthesis, a crucial function during chloroplasts biogenesis (Carol et al., 1999; Aluru et al., 2001; Shahbazi et al., 2007). In any case, chlororespiration has always been considered a complement of photosynthesis, being only an element of a large network of factors involved in stress tolerance and photosynthesis regulation (Foudree et al., 2012).

The study of respiration in non-photosynthetic plastids has received less attention. Nevertheless, growing evidence has accumulated about the presence of some respiratory components in chromoplasts, etioplasts, and amyloplasts of different plant species. As a consequence, a more global role of PTOX in plastid metabolism has been suggested (Aluru et al., 2001; Morstadt et al., 2002; Barr et al., 2004; Nixon and Rich, 2006; McDonald et al., 2011; Foudree et al., 2012). Recently, two studies have provided convincing evidence about the existence of an active respiratory chain in tomato fruit chromoplasts linked to ATP synthesis (Pateraki et al., 2013; Renato et al., 2014). This article aims to review recently published results regarding the presence of respiratory activity in non-photosynthetic plastids and to identify the major gaps that need to be addressed in future research projects.

## Respiration in Non-Photosynthetic Plastids

### Chromoplasts

Chromoplasts are plastids specialized in the biosynthesis and accumulation of carotenoids. They are found in flowers, fruits, and roots, conferring to these plant tissues their characteristic red, orange, or yellow color. They are originated through the differentiation of other plastids, mainly chloroplasts and amyloplasts (Camara et al., 1995; Li and Yuan, 2013). Among non-photosynthetic plastids, chromoplasts are the most studied since carotenoids are relevant for the nutritional and organoleptic quality of many agricultural products (Li and Yuan, 2013).

The first hints suggesting the presence of a respiratory pathway in chromoplasts were obtained through the study of phytoene desaturase (PDS), an enzyme involved in carotenoid biosynthesis. PDS catalyzes two consecutive dehydrogenation reactions of phytoene and transfers the electrons to PQ (Norris et al., 1995). In daffodil (*Narcissus pseudonarcissus*) chromoplasts, Mayer et al. (1992) and Beyer et al. (1994) proposed the existence of enzymatic activities which regulate the redox estate of PQs in darkness, using NADH and NADPH as electron donors and oxygen as final acceptor. Nievelstein et al. (1995) reported an oxygen consumption activity in daffodil chromoplast membranes, which was dependent on NAD(P)H and sensitive to respiratory uncouplers, suggesting that this respiration could generate membrane proton gradients. This work was the first to define chromorespiration as a respiratory redox pathway in chromoplast membranes linked to phytoene desaturation. Later, it was described that liposomes containing chromoplast proteins and energized with an acid-base transition were able to produce ATP, suggesting that daffodil chromoplasts contain a functional H^+^-ATP synthase complex (Morstadt et al., 2002).

Further studies brought molecular support to the enzymatic activities attributed to chromorespiration. The PTOX was found in chromoplasts from tomato (*Solanum lycopersicum*) and bell pepper (*Capsicum annuum*) fruits (Josse et al., 2000). In both species, PTOX expression increases during ripening, paralleling PDS expression and the differentiation of chloroplasts into chromoplasts. Thus, PTOX was proposed to participate in carotenoid desaturation in ripening fruits (Josse et al., 2000). The tomato *ghost* mutant is impaired in PTOX gene and is equivalent to the *Arabidopsis* mutant *immutans*. The *ghost* phenotype is similar to PDS-deficient mutants and, as a consequence, PTOX was considered a PDS cofactor (Josse et al., 2000; Barr et al., 2004).

Recent proteomic studies have reported the presence of several proteins related to electron transport and ATP synthesis in chromoplasts. Subunits of ATP synthase, cytochrome b_6_f complex and Ndh are present in chromoplasts from tomato fruits (Barsan et al., 2010). Moreover, when comparing the proteome of fruit plastids at different ripening stages, it was found that some electron carriers and the ATP synthase subunits are maintained at significant levels in red tomatoes (Barsan et al., 2012). This was also confirmed in chromoplasts from watermelon (*Citrullus lanatus*), carrot (*Daucus carota*), orange cauliflower (*Brassica oleracea*), red papaya (*Carica papaya*), red bell pepper, and sweet orange (*Citrus sinensis*) (Zeng et al., 2011; Wang et al., 2013). Interestingly, these electron carriers and ATP synthase subunits are present even when chromoplasts are not originated from chloroplasts and they have never performed photosynthesis, like in carrots or in watermelon and orange pulp.

On the other hand, a large number of proteins involved in carbohydrate metabolism were found in chromoplasts of several species, for instance some enzymes of glycolysis and the pentose phosphate pathway, translocators of triose-phosphate, glucose-6-phosphate, adenine nucleotides, etc. (Siddique et al., 2006; Zeng et al., 2011; Barsan et al., 2012; Wang et al., 2013). Also, it has been described that isolated chromoplasts are able to synthesize large amounts of lipids without external supply of ATP (Angaman et al., 2012). All these findings suggest an important activity of energy production within chromoplasts and points out a more general role of chromorespiration beyond its contribution to carotenoid desaturation.

Chromoplastic ATP synthesis was further evidenced when an atypical isoform of the ATP synthase complex was identified in tomato fruit chromoplasts (Pateraki et al., 2013). This ATP synthase contains a specific γ-subunit (γ_2_) which increases its expression during ripening and is absent in green tissues. The silencing of this subunit caused an inhibition of chromoplast ATP synthesis (Pateraki et al., 2013). In photosynthetic tissues, the γ-subunit (γ_1_) of ATP synthase has a regulatory role. It contains a dithiol domain which provides a redox switch to inactivate the complex in dark conditions, preventing ATP hydrolysis (Samra et al., 2006). However, the γ_2_ isoform does not have the cysteine residues of the dithiol domain, suggesting that the ATP synthase complex is always active. This atypical γ_2_-subunit is also found in plastids from other non-photosynthetic tissues, like roots (Kohzuma et al., 2012). It is possible that the γ_2_-subunit has evolved to work efficiently in the physiological conditions of non-photosynthetic plastids, which may present lower electrochemical potentials than chloroplasts (Pateraki et al., 2013).

Respiration and ATP synthesis were quantified in isolated tomato fruit chromoplasts using NAD(P)H as electron donors (Renato et al., 2014). It was found that octyl gallate, an inhibitor of PTOX, prevented both respiration and ATP synthesis, confirming experimentally that PTOX is involved in chromorespiration (Renato et al., 2014). Also, the use of specific inhibitors suggested the participation of two different NAD(P)H dehydrogenases and the cytochrome b_6_f complex. Moreover, the existence of proton gradients through chromoplast membranes was evidenced by the study of proton uncouplers and sonicated chromoplasts (Renato et al., 2014).

#### A Model for Chromorespiration

**FIGURE 1:**
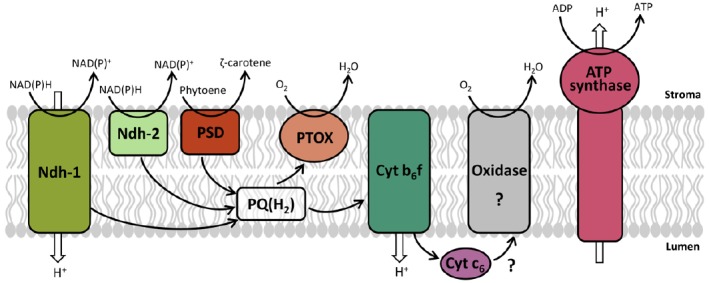
**Model proposed for chromorespiration.** The electron transport chain is located in the inner membranes of chromoplasts, and the electron entrance through NADH and NADPH takes place by the stromal side. The electron carriers shown are: Ndh-1, NAD(P)H dehydrogenase complex; Ndh-2, type II NAD(P)H dehydrogenase; PDS, phytoene desaturase; PTOX, plastid terminal oxidase; Cyt b_6_f, cytochrome b_6_f complex; Cyt c_6_, cytochrome c_6_. A possible cytochrome c oxidase or similar (Oxidase) is proposed. ATP synthase complex is also shown. Electron transfer reactions are indicated by black arrows and proton movement across membrane by white arrows.

Even though the components of the chromorespiratory pathway are still unclear, we propose a preliminary model with the aim of summarizing the available data and suggesting future research (Figure 1). The electron transport chain is probably located in the inner membranes of chromoplasts, which form elongated sacs or convoluted compartments (Renato et al., 2014). Both NADH and NADPH transfer electrons to the oxidized PQ pool probably through the Ndh, which is similar to the mitochondrial complex I and is able to pump protons across membranes. Ndh is present in chromoplasts and its dysfunction severely affects fruit ripening (Nashilevitz et al., 2010), so it could play a significant bioenergetic role. However, type II NAD(P)H dehydrogenase may also participate in chromorespiration (Renato et al., 2014). Type II dehydrogenases are monomeric enzymes without proton pumping activity which are targeted to mitochondria, plastids, and peroxisomes (Carrie et al., 2008). The presence of these enzymes in chromoplasts has not been yet tested, and further studies are needed to clarify this issue. Besides, the PQ pool is also reduced by PDS, which transfers the electrons resulting from the desaturation steps of phytoene during carotenoid biosynthesis (Norris et al., 1995).

The reduced PQ pool (PQH_2_) may transfer the electrons directly to PTOX, the terminal oxidase which is responsible of the oxygen consumption activity in chromoplasts (Renato et al., 2014). Alternatively, the electrons could pass through the cytochrome b_6_f complex, adding a supplementary proton pumping site. This hypothesis is supported by the marked effect of a cytochrome b_6_f inhibitor in chromoplast ATP synthesis (Renato et al., 2014). However, the electron acceptor of the cytochrome b_6_f is unknown. Plastocyanin plays this role in chloroplasts, but it is only found in photosynthetic tissues (Wastl et al., 2002). An alternative electron acceptor could be the cytochrome c_6_. This cytochrome is present in cyanobacteria and is involved in the interconnection of respiratory and photosynthetic chains: it takes electrons from cytochrome b_6_f and transfers them to photosystem I or to cytochrome c oxidases (Ho et al., 2011). Cytochrome c_6_ is also present in higher plants, although its function is not yet known (Wastl et al., 2002). In tomato fruit, cytochrome c_6_ expression increases during ripening, paralleling PTOX expression and chromoplast differentiation (Renato et al., 2014), suggesting that it may participate in chromorespiration. Further experimental evidences are needed to clarify this point.

As mentioned above, in cyanobacteria the cytochrome c_6_ can play a respiratory role and transfer the electrons to a cytochrome c oxidase (Hart et al., 2005; Ho et al., 2011). Several proteomic studies have reported the presence of some subunits of cytochrome c oxidase in chromoplasts from tomato fruit (Barsan et al., 2010, 2012; Wang et al., 2013), orange fruit (Zeng et al., 2011), watermelon, carrot, papaya, and bell pepper (Wang et al., 2013). This result may be interpreted as mitochondrial contamination. However, it has been obtained repeatedly in independent studies, so it is possible that chromoplasts could contain a plastidial form of a cytochrome c oxidase. This oxidase could have been inherited from cyanobacteria ancestor and could participate in chromorespiration oxidizing the cytochrome c_6_ (Figure 1). Nevertheless, more studies are required to elucidate this issue.

One or several components of the electron transport chain in chromoplasts pump protons across membranes (Figure 1) and the proton gradients generated are used by the plastid ATP synthase. Therefore, chromorespiration is a respiratory process that works similarly to the oxidative phosphorylation of mitochondria, where there is an electron transfer from electron donors (NADH and/or NADPH) to an electron acceptor (oxygen) coupled to proton pumping and chemiosmotic ATP synthesis.

### Other Non-Photosynthetic Plastids

To our knowledge, dark respiratory processes in non-photosynthetic plastids other than chromoplasts have not been reported. However, published data suggest the presence of several electron carriers and subunits of ATP synthase in etioplasts and amyloplasts. Etioplasts are differentiated from proplastids in photosynthetic tissues grown in darkness. They are characterized by the accumulation of protochlorophyllide (chlorophyll precursor) and the presence of paracrystalline prolamellar bodies (Plöscher et al., 2011). Although etioplasts are a special type of plastid related to chloroplasts, we include them in this work because they could present a light-independent respiratory process when photosynthesis is not yet operative. Amyloplasts are specialized in the synthesis and accumulation of starch (Andon et al., 2002). We were not able to find any information regarding respiratory components in any other non-photosynthetic plastid, like elaioplasts and proteoplasts (specialized in lipid and protein accumulation, respectively).

Proteomic studies have revealed high levels of Ndh in etioplasts from barley (*Hordeum vulgare*, Catala et al., 1997; Guera et al., 2000), pea (*Pisum sativum*, Lennon et al., 2003), *Arabidopsis* (Peng et al., 2008), tobacco (*Nicotiana tabacum*, Karcher and Bock, 2002), corn (*Zea mays*), and rice (*Oryza sativa*) (Fischer et al., 1997). Its accumulation is independent of light and decreases during greening, suggesting a possible role in membrane energization when the photosynthetic electron transport chain is not yet working (Guera et al., 2000). In chloroplasts, the Ndh is assembled with PSI forming a supercomplex (Peng and Shikanai, 2011). Etioplasts are devoid of PSI and the Ndh is found as an independent functional unit (Peng et al., 2008), so its composition could be different in chloroplasts and etioplasts. Regarding this possibility, it has been reported that some subunits of the Ndh complex are modified through post-transcriptional editing. Particularly, the transcript of the ndhB subunit is altered in one nucleotide in photosynthetic tissues, but it is not edited in etiolated tissues, and as a result two different isoforms of this subunit are synthesized in chloroplasts and etioplasts (Karcher and Bock, 2002). Interestingly, this lack of transcript editing in ndhB also happens in tomato fruit chromoplasts (Kahlau and Bock, 2008).

The cytochrome b_6_f complex is present in etioplasts from pea and barley (Kanervo et al., 2008; Reisinger et al., 2008; Plöscher et al., 2011). Also, one of its subunits has been reported in proteomic analysis of wheat (*Triticum aestivum*) endosperm amyloplasts (Dupont, 2008).

Several subunits of ATP synthase have been found in significant amounts in etioplasts from pea, corn, *Arabidopsis*, rice, and barley (Fischer et al., 1997; Kanervo et al., 2008; Plöscher et al., 2011). The α-subunit has also been detected in amyloplasts from wheat endosperm (Andon et al., 2002; Balmer et al., 2006; Dupont, 2008). In addition, the γ_2_-subunit of the ATP synthase, which participates in chromorespiration, is predominantly expressed in *Arabidopsis* roots. A knockout of this gene affected root morphology, suggesting that it plays an important role in non-photosynthetic tissues containing amyloplasts (Kohzuma et al., 2012).

The PTOX is ubiquitously expressed in *Arabidopsis* and tomato (Aluru et al., 2001; Barr et al., 2004), including tissues containing etioplasts and amyloplasts such as etiolated cotyledons and roots. Also, the ultrastructure of etioplasts and amyloplasts is severely affected when PTOX is not functional (Aluru et al., 2001), indicating that some processes in these plastids depend on PTOX activity. Moreover, PTOX expression is strongly upregulated in etioplasts during the de-etiolation of dark-grown seedlings, according to the reported accumulation of carotenoids in this transition (Rodríguez-Villalón et al., 2009).

Therefore, proteomic and genomic data provide several hints about the presence of a respiratory electron transport chain in etioplasts and amyloplasts which could be linked to ATP synthesis. However, further studies are required to better understand its biological function and to identify all the electron carriers involved in this activity.

## Conclusions and Future Directions

Dark respiration is one of the less understood processes in plastids. Chlororespiration is mainly considered a mechanism to adapt photosynthesis to changing environmental conditions. Recently, chromorespiration has been shown to be linked to chemiosmotic ATP synthesis, and proteomic studies suggest that a similar respiratory process may be active in etioplasts and amyloplasts.

Future studies should be conducted to address the large number of open questions regarding respiratory processes in chromoplasts and other non-green plastids. For instance, the source of the electron donors NADPH and NADH is unknown. They could be generated inside the plastids or imported from the cytosol instead. Also, the physiological role of the ATP generated by chromorespiration is not known. In ripe tomato, PTOX activity is responsible of one quarter of total fruit oxygen consumption and contribute significantly to tissue ATP content (Renato et al., 2014). It would be interesting to obtain more data about plastid respiration and ATP synthesis in other plant tissues containing chromoplasts or amyloplasts. Further understanding of respiratory processes in these plastids could be useful to overcome limitations in the biosynthesis of carotenoids and starch, and thus to improve the quality of several agricultural products. Besides, further studies about etioplast respiration could provide new data regarding the etiolation and de-etiolation transitions during germination.

### Conflict of Interest Statement

The authors declare that the research was conducted in the absence of any commercial or financial relationships that could be construed as a potential conflict of interest.
